# Case Report: Ischemic stroke revealing carotid fibromuscular dysplasia with multiple metabolic disturbances in a 42-year-old man

**DOI:** 10.3389/fcvm.2026.1828224

**Published:** 2026-09-08

**Authors:** Min Li, Yinbao Hu

**Affiliations:** Department of Neurology, Yantai Yuhuangding Hospital Affiliated to Qingdao University, Yantai, Shandong, China

**Keywords:** case report, diabetes mellitus, digital subtraction angiography, fibromuscular dysplasia, hypertriglyceridemia, internal carotid artery, ischemic stroke, male

## Abstract

**Background:**

Fibromuscular dysplasia (FMD) is a rare, non-atherosclerotic, non-inflammatory vascular disease that predominantly affects women and can lead to stenosis, dissection, aneurysm, or occlusion of medium-sized arteries. Cerebrovascular FMD is an important but underrecognized cause of ischemic stroke, particularly in young to middle-aged adults.

**Case presentation:**

A 42-year-old man presented with acute-onset slurred speech and right-sided weakness. Brain magnetic resonance imaging (MRI) was precluded by claustrophobia, but serial non-contrast head CT scans (admission and 48-hour) confirmed an acute ischemic infarct in the left basal ganglia. Intravenous thrombolysis with alteplase was administered within the therapeutic window. Cervicocerebral computed tomography angiography (CTA) revealed a typical “string-of-beads” sign in the left internal carotid artery (L-ICA) C1 segment, suggestive of FMD, which was subsequently confirmed by digital subtraction angiography (DSA). Subsequent laboratory workup revealed multiple metabolic abnormalities: severe hypertriglyceridemia (17.54 mmol/L), hyperuricemia (569 μmol/L), elevated liver enzymes (ALT 152 U/L, AST 128 U/L), and newly diagnosed diabetes mellitus (HbA1c 7.2%). Extensive autoimmune and thrombophilia panels were negative. DSA demonstrated the characteristic “string-of-beads” appearance in the mid-cervical segment of the L-ICA, pathognomonic for the medial fibroplasia subtype of FMD. The patient was managed with dual antiplatelet therapy, high-intensity statin, blood pressure control and risk factor modification. He showed gradual neurological improvement and was discharged on day 14 with a modified Rankin Scale score of 1. At the 3-month follow-up, his neurological function had fully recovered (mRS 0), with no recurrent events. Blood pressure was well-controlled, and laboratory tests showed normalized lipid and glucose profiles. Renal artery duplex ultrasound at 3 months revealed no evidence of FMD.

**Conclusion:**

This case highlights that FMD should be considered in the differential diagnosis of ischemic stroke in middle-aged patients, including men, even in the presence of traditional vascular risk factors. The coexistence of FMD with multiple metabolic disturbances in this case, while likely coincidental, underscores the complexity of stroke etiology and the importance of a comprehensive diagnostic approach that does not prematurely attribute stroke to common risk factors alone. Carotid FMD, though rare, is a treatable cause of stroke, and early diagnosis via DSA can guide appropriate management to prevent recurrence.

## 1 Introduction

Fibromuscular dysplasia (FMD) is an idiopathic, segmental, non-inflammatory, and non-atherosclerotic disease of the arterial wall, most commonly affecting the renal and cervicocranial arteries (1). It is recognized as a rare disease, with an estimated prevalence in the general population as low as 0.02% based on autopsy studies of the cervical internal carotid arteries (2). However, among patients undergoing cerebrovascular angiography for various indications, the prevalence ranges from 0.3% to 6.8% (3). FMD disproportionately affects women, accounting for 86%–90% of diagnosed cases in large registries (4, 5).

**Table 1:** Clinical timeline of key events.

Time Point	Clinical Events & Findings	Interventions
Day 0 (Index event, 3 h post-onset)	Acute onset of slurred speech and right-sided weakness (NIHSS 3). BP 170/100 mmHg. Initial head CT negative for hemorrhage.	Admission to Emergency Department.
Day 0 (3.5 h post-onset)	Confirmed acute ischemic stroke based on clinical deficits and negative initial CT.	Intravenous thrombolysis (Alteplase 0.9 mg/kg).
Day 0–1	Persistent hypertension (systolic >170 mmHg).	IV nicardipine and urapidil for blood pressure control.
Day 2	Follow-up head CT confirmed left basal ganglia infarct. L-ICA “string-of-beads” sign detected on CTA.	Started dual antiplatelet therapy (Aspirin + Clopidogrel).
Day 11	DSA confirmed medial fibroplasia subtype of FMD in L-ICA.	Transitioned to oral antihypertensives (olmesartan, nifedipine, metoprolol).
Day 14	Neurological deficits improved (mRS 1). BP stabilized at 130–140/80–90 mmHg.	Discharged with medications and lifestyle counseling.
Month 3	Complete neurological recovery (mRS 0). Labs normalized. Renal artery duplex normal.	Maintained on aspirin 100 mg daily and rosuvastatin 10 mg daily.

Cerebrovascular FMD is a significant cause of stroke, particularly in younger populations. A recent 2023 retrospective cohort study found that stroke or transient ischemic attack (TIA) was the presenting symptom in up to 63% of patients with cerebrovascular FMD (6). Furthermore, FMD is identified in approximately 15% of patients with spontaneous cervical artery dissection, a major cause of stroke in the young (7). Despite these associations, FMD remains underdiagnosed due to a lack of clinical awareness and the nonspecific nature of its presenting symptoms.

This case is particularly noteworthy because: (1) it occurs in a male patient, reminding us that while FMD is female-predominant, it can affect men; (2) it demonstrates the coexistence of FMD with multiple metabolic risk factors (newly diagnosed diabetes, severe hypertriglyceridemia, hyperuricemia, and hepatic dysfunction), a scenario that is rarely reported; and (3) it highlights the importance of considering rare stroke etiologies even when common risk factors are present.

## 2 Case presentation

### 2.1 Patient information and initial presentation

A 42-year-old man presented to the emergency department with a 3-hour history of acute-onset slurred speech and right-sided weakness. His past medical history was notable for hypertension diagnosed 3 years prior, for which he had not been taking regular antihypertensive medication. He had no history of diabetes, hyperlipidemia, prior stroke, or cardiovascular disease. He was a non-smoker, reported no illicit drug use, and had no family history of stroke or connective tissue disorders.

### 2.2 Clinical examination

On admission, his blood pressure was 170/100 mmHg, heart rate 96 bpm, and oxygen saturation 98% on room air. Neurological examination revealed an alert and oriented patient with mild dysarthria. Cranial nerve assessment showed flattening of the right nasolabial fold and tongue deviation to the right. Motor testing demonstrated right upper extremity weakness (grade 4/5), with normal strength in the other limbs. Finger-to-nose testing revealed right-sided dysmetria, while sensory examination to light touch and pinprick was intact. Deep tendon reflexes were symmetric and plantar responses flexor bilaterally. His National Institutes of Health Stroke Scale (NIHSS) score on admission was 3.

### 2.3 Initial investigations and management

**Figure 1:**
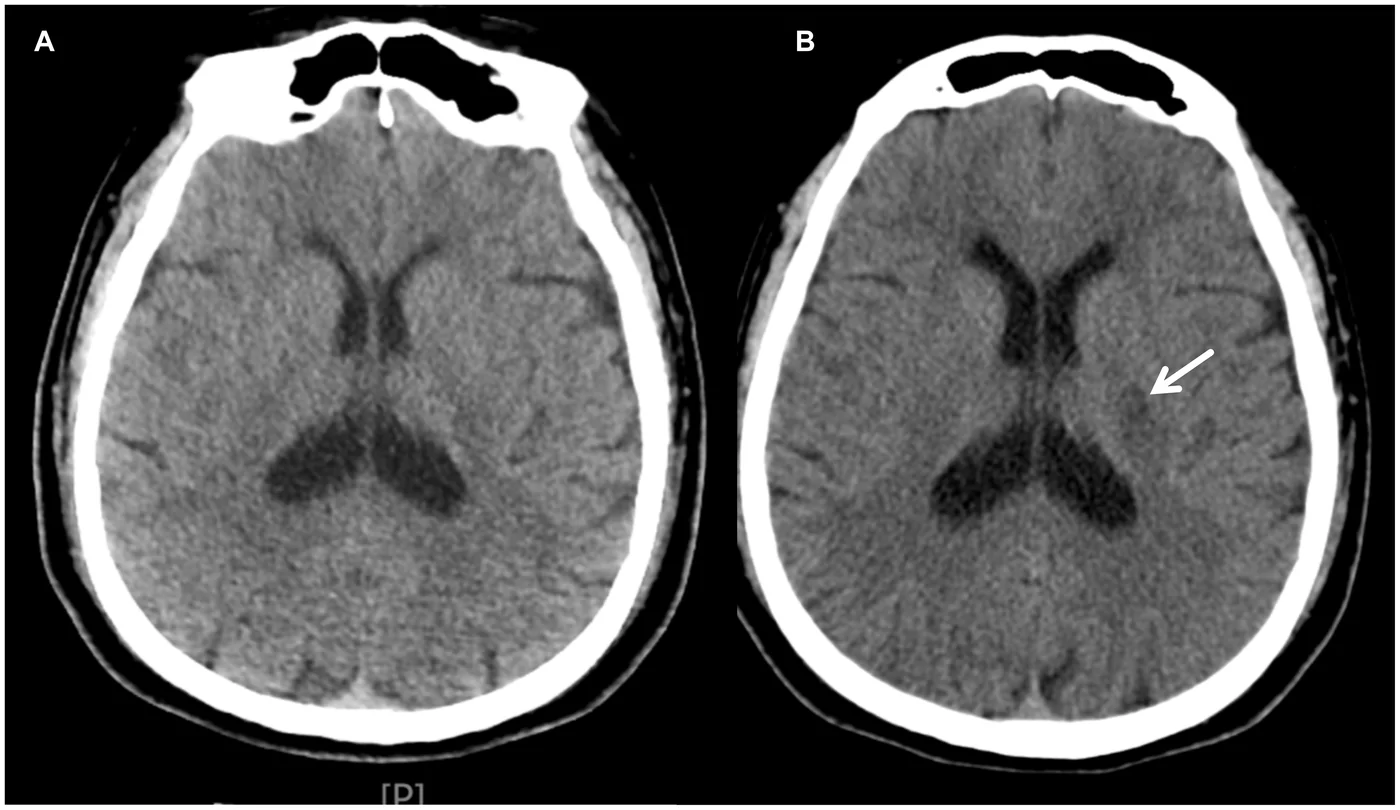
Serial non-contrast cranial computed tomography (CT) scans of the brain (axial views). **(A)** Initial non-contrast head CT at admission (3 h after symptom onset) shows a subtle focal hypodense area in the left basal ganglia region, without intracranial hemorrhage or mass effect. **(B)** Follow-up non-contrast head CT (48 h after symptom onset) demonstrates a well-demarcated hypodense infarct (white arrow) in the same left basal ganglia territory with increased lucency compared with the initial scan. Brain magnetic resonance imaging (MRI) could not be performed due to the patient's severe claustrophobia; the serial CT comparison confirms the location and evolution of the acute ischemic infarct, providing critical imaging evidence for the diagnosis and therapeutic decision-making of acute ischemic stroke.

Non-contrast head CT on admission showed no evidence of intracranial hemorrhage or early ischemic changes (Figure 1A). Intravenous thrombolysis with alteplase (0.9 mg/kg) was administered starting 3.5 h after symptom onset. Brain MRI with diffusion-weighted imaging was planned but could not be performed due to the patient's severe claustrophobia; therefore, the diagnosis of acute ischemic stroke was confirmed by serial non-contrast CT scans (admission and 48-hour), which demonstrated the evolution of a hypodense lesion in the left basal ganglia consistent with the patient's clinical deficits (Figure 1B). Electrocardiogram showed sinus tachycardia without ischemic changes. Laboratory findings on admission revealed the following selected abnormalities: potassium 2.72 mmol/L (reference: 3.5–5.1), glucose 10.73 mmol/L (reference: 3.9–6.1), HbA1c 7.2% (reference: <5.7%), total cholesterol 7.58 mmol/L (reference: <5.2), triglycerides 17.54 mmol/L (reference: <1.7), LDL**-**C 1.13 mmol/L (reference: <3.4), uric acid 569 μmol/L (reference: 208–428), and elevated liver enzymes (ALT 152 U/L, AST 128 U/L, GGT 163 U/L); additional inflammatory markers were within normal limits (CRP < 3 mg/L, reference: <10 mg/L; ESR 12 mm/h, reference: <20 mm/h). Hypokalemia was corrected with intravenous and oral potassium supplementation. The markedly elevated triglycerides and HbA1c indicated newly diagnosed diabetes mellitus with severe hypertriglyceridemia, while elevated liver enzymes suggested possible nonalcoholic fatty liver disease.

### 2.4 Hospital course and further workup

#### 2.4.1 Days 2–5

The patient experienced fluctuating blood pressure and intermittent episodes of chest tightness. A follow-up non-contrast head CT at 48 h post-symptom onset showed a well-demarcated hypodense infarct in the left basal ganglia (Figure 1B), confirming the diagnosis of acute ischemic stroke given that MRI was not possible due to claustrophobia. Pulmonary embolism was ruled out by pulmonary CTA. Coronary CTA revealed a non-obstructive calcified plaque in the left anterior descending artery (approximately 40% stenosis) and a myocardial bridge. Autoimmune and thrombophilia workup, including ANA, ANCA, antiphospholipid antibodies, and lupus anticoagulant, was negative. Given the normal CRP and ESR, active vasculitis was considered unlikely.

**Figure 2:**
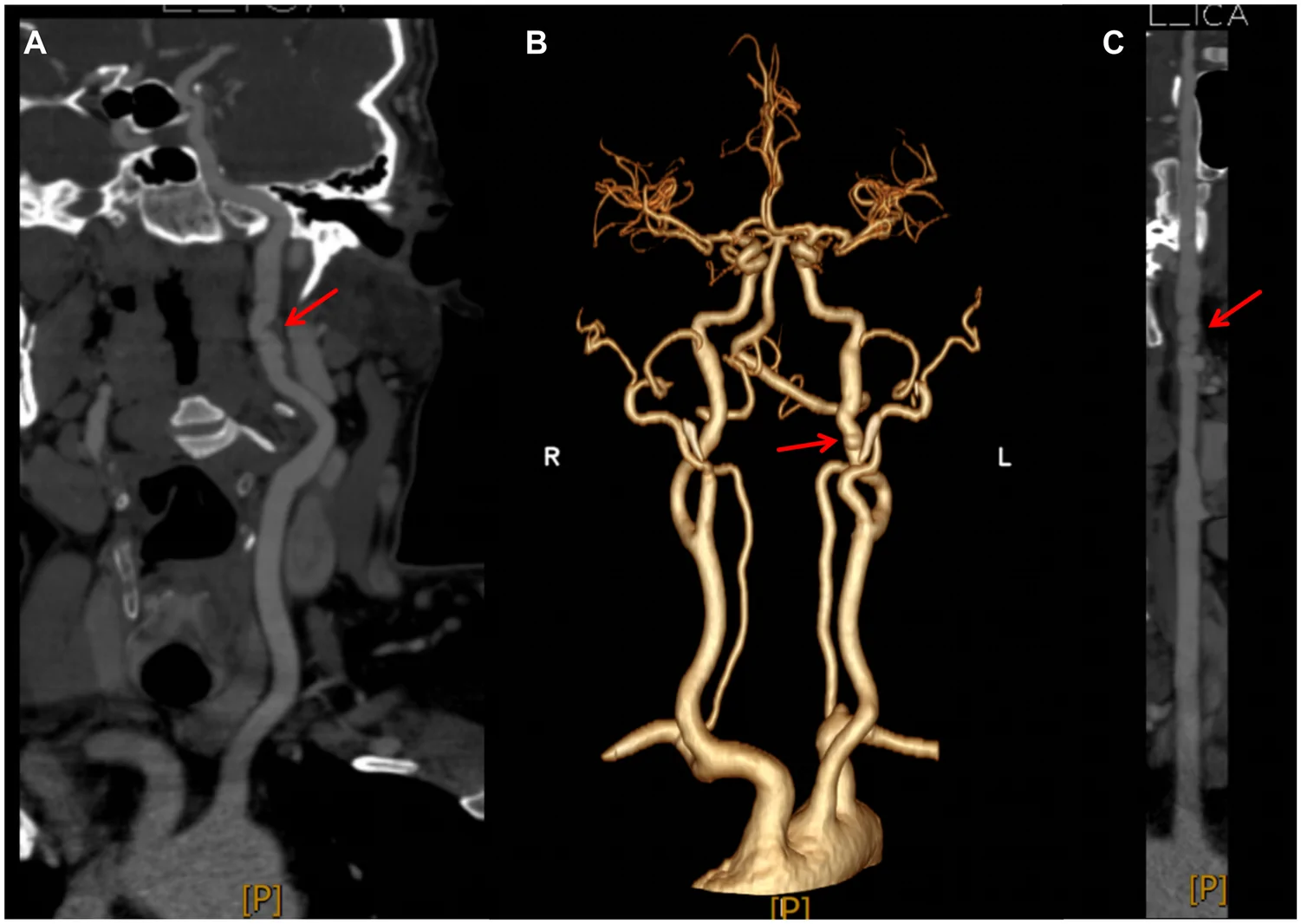
Cervicocerebral computed tomography angiography (CTA) of the left internal carotid artery (L-ICA). **(A–C)** Multiplanar reconstructions of cervical CTA demonstrate the characteristic “string-of-beads” appearance in the mid-cervical C1 segment of the left internal carotid artery (red arrows), characterized by alternating segments of focal arterial stenosis and post-stenotic dilatation. This typical angiographic finding on CTA is highly suggestive of fibromuscular dysplasia (FMD), and the multi-view imaging clearly depicts the spatial distribution of the abnormal vascular segment without obvious atherosclerotic plaque or vascular dissection in the surrounding vessels.

Cervicocerebral CTA with multiplanar reconstruction demonstrated a classic “string-of-beads” appearance with alternating stenosis and dilatation in the mid-cervical C1 segment of the left internal carotid artery (L-ICA) (Figures 2A–C), highly suggestive of FMD. No atherosclerotic plaque, vascular dissection, or aneurysm was found in the abnormal segment or surrounding vessels. The right internal carotid artery and vertebral arteries showed no significant stenosis. Chest and aortic CTA showed no evidence of aortic pathology, dissection, or large vessel vasculitis.

#### 2.4.1 Day 11

**Figure 3:**
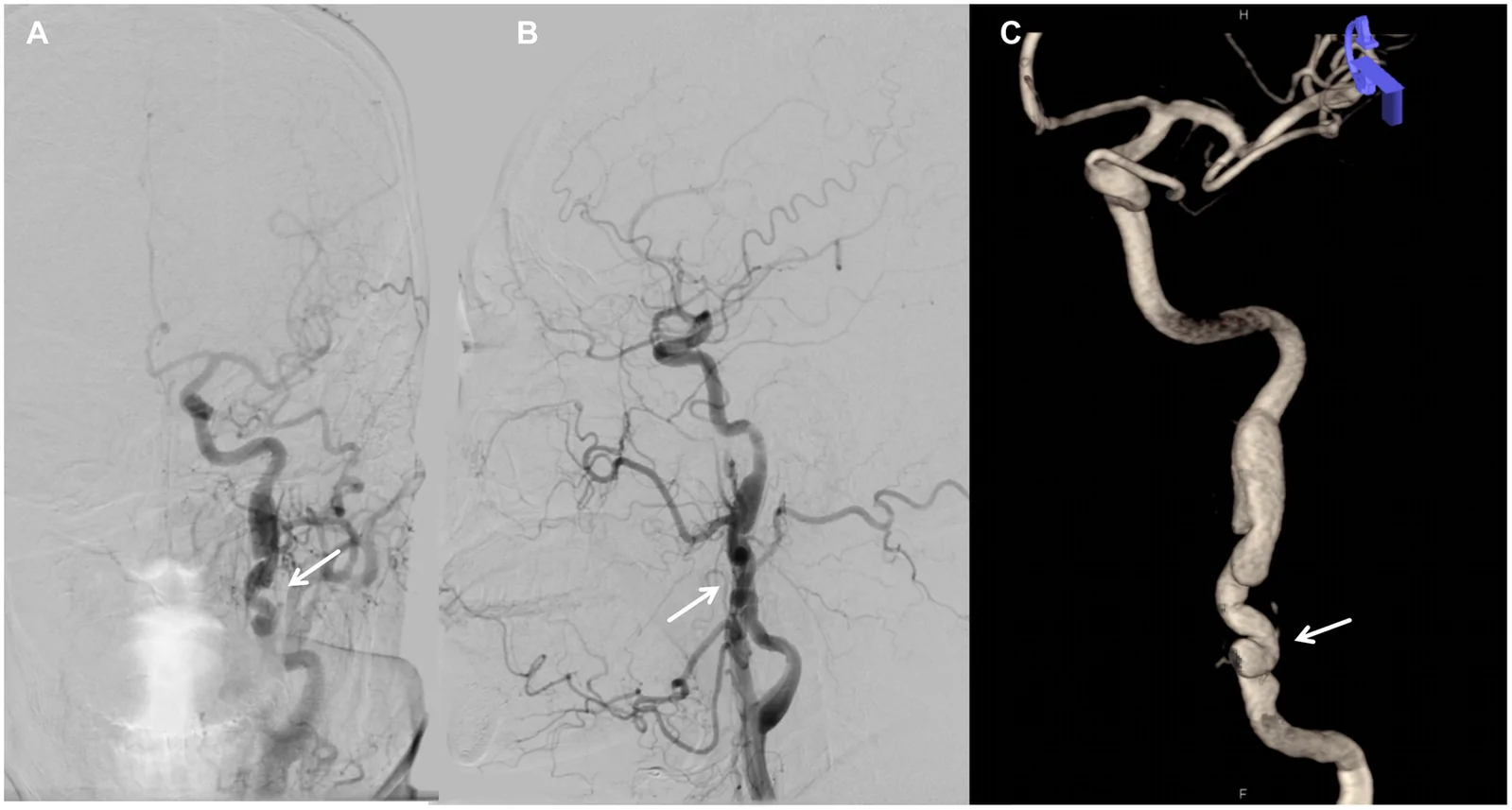
Digital subtraction angiography (DSA) of the left internal carotid artery (L-ICA). **(A)** Anteroposterior view demonstrating the classic “string-of-beads” appearance (white arrow) in the mid-cervical C1 segment, characterized by alternating focal stenoses and post-stenotic dilatations. **(B)** Lateral view confirming the same “string-of-beads” morphology (white arrow) in the affected segment. **(C)** Three-dimensional reconstruction providing an overview of the spatial configuration of the diseased arterial segment (white arrow) and its relationship with adjacent vascular structures. This angiographic finding is pathognomonic for the medial fibroplasia subtype of fibromuscular dysplasia (FMD).

Digital Subtraction Angiography (DSA) confirmed the diagnosis, revealing classic alternating areas of stenosis and dilatation (“string-of-beads” sign) involving the mid-cervical C1 segment of the L-ICA (Figure 3), pathognomonic for the medial fibroplasia subtype of FMD. The right ICA and vertebral arteries showed no significant stenosis, dissection, or aneurysm.

### 2.5 Diagnosis

Based on the integration of clinical presentation, laboratory findings, and characteristic vascular imaging, the final diagnoses were established as acute ischemic stroke of the left basal ganglia secondary to left internal carotid artery fibromuscular dysplasia (medial fibroplasia type), with coexisting hypertension (grade 3, very high risk), newly diagnosed type 2 diabetes mellitus, severe hypertriglyceridemia, hyperuricemia, suspected nonalcoholic fatty liver disease, and hypokalemia that resolved with supplementation. Although the patient presented with multiple traditional vascular risk factors, the ipsilateral location of the FMD lesion, the absence of other identifiable embolic sources (including atrial fibrillation, aortic arch atheroma, and intracranial large-artery stenosis), and the exclusion of active vasculitis by normal inflammatory markers (CRP, ESR) and negative autoimmune serologies collectively supported FMD as the primary etiological mechanism underlying the index stroke. However, a concurrent contribution from hypertensive small-vessel disease cannot be entirely excluded; therefore, the stroke mechanism is best interpreted as likely multifactorial, with FMD serving as the predominant driver.

### 2.6 Treatment and outcome

The patient was started on dual antiplatelet therapy (aspirin 100 mg and clopidogrel 75 mg daily) for secondary stroke prevention, given the diagnosis of FMD-related stroke without dissection. The short-term dual antiplatelet regimen was chosen in accordance with acute stroke guidelines for non-cardioembolic ischemic stroke, with the intention to transition to lifelong single antiplatelet therapy after 21 days. High-intensity statin therapy (rosuvastatin 10 mg daily) was initiated, with ezetimibe 10 mg added for additional lipid lowering. It should be emphasized that statin therapy was primarily prescribed for the treatment of the patient's severe hypertriglyceridemia and elevated total cholesterol, rather than as a specific disease-modifying therapy for FMD. Antihypertensive therapy was transitioned to oral medications (olmesartan 80 mg, nifedipine 30 mg, and metoprolol 47.5 mg daily). Newly diagnosed diabetes was managed with dietary counseling and glucose monitoring; no hypoglycemic agents were required at discharge. Hepatoprotective agents were administered for elevated liver enzymes.

His neurological deficits gradually improved. By discharge on day 14, his right-sided weakness had resolved to grade 5, though mild right-sided dysmetria persisted. The modified Rankin Scale (mRS) score at discharge was 1. Blood pressure was controlled at 130–140/80–90 mmHg. He was counseled on lifestyle modifications, medication adherence, and the importance of regular follow-up.

### 2.7 Follow-up at 3 months

The patient returned for outpatient follow-up 3 months after discharge. He reported no further neurological symptoms and had returned to full-time work. Neurological examination was entirely normal, with no residual weakness or dysmetria. His modified Rankin Scale (mRS) score had improved to 0, indicating a complete recovery.

Blood pressure was well-controlled at 110/80 mmHg. Fasting lipid profile and glucose levels were within normal limits on aspirin and statin therapy. Renal artery duplex ultrasound, performed as recommended for all patients with cervicocranial FMD, showed normal renal arteries with no evidence of FMD. However, duplex ultrasound is a screening tool rather than a definitive diagnostic modality; therefore, renal artery CTA or MRA has been scheduled at the 1-year follow-up to definitively exclude renal artery FMD. Intracranial aneurysm screening via MRA or CTA was not performed during the acute hospitalization because the patient had no clinical indicators (e.g., headache, family history of aneurysms) that would mandate immediate invasive investigation; however, such screening has been scheduled for the 1-year follow-up as part of comprehensive vascular surveillance. He remained on aspirin 100 mg daily and rosuvastatin 10 mg daily, with no recurrent strokes or TIAs during the follow-up period. A summary of the clinical timeline is presented in Table 1.

## 3 Discussion

We report a case of acute ischemic stroke in a 42-year-old man secondary to fibromuscular dysplasia of the left internal carotid artery, with the classic “string-of-beads” appearance on DSA. This case is instructive for several reasons: (1) it demonstrates the pathognomonic angiographic findings of FMD; (2) it occurs in a male patient, challenging the stereotype of FMD as a disease exclusively affecting women; (3) it highlights the complex interplay between FMD and multiple metabolic disturbances; and (4) it underscores the importance of considering rare stroke etiologies even when common risk factors are present.

### 3.1 FMD as a cause of ischemic stroke

Cerebrovascular FMD is an established but often overlooked cause of ischemic stroke. The pathognomonic “string-of-beads” appearance seen on DSA in our patient corresponds to the medial fibroplasia subtype, which accounts for 80%–90% of all FMD cases (1). This subtype is characterized by alternating areas of stenosis and dilation due to fibromuscular ridges and thinning of the media, creating a beaded appearance that is wider than the normal artery (8). In clinical practice, cervicocerebral CTA is a valuable non-invasive screening tool for FMD, and when it reveals the characteristic “string-of-beads” sign, it can guide the need for confirmatory DSA, as demonstrated in this case. The stroke mechanism in FMD is likely thromboembolic, arising from the diseased arterial segment, although hemodynamic compromise from severe stenosis can also contribute (9). In our patient, the left basal ganglia infarct makes artery-to-artery embolism the most likely mechanism.

The prevalence of FMD in the general population is low, but its impact on stroke risk is significant. In a large US inpatient database study, 63.6% of hospitalized FMD patients had cerebrovascular disease, and 35.7% had stroke (4). A study by Harriott et al. found that among 86 patients with cerebrovascular FMD, stroke or TIA was the initial presentation in 63%, and during a median follow-up of 47 months, 23% experienced recurrent cerebrovascular events (6). These data underscore the importance of accurate diagnosis and secondary prevention in this population.

### 3.2 FMD in men: a reminder of epidemiological diversity

Although FMD is well-documented to predominantly affect women (86%–90% in large registries), it does occur in men (4, 5). A 2022 analysis of the ARCADIA registry reported that male patients with FMD tend to present at a younger age and have a higher prevalence of vascular risk factors compared to female patients (10). Our case aligns with this observation: our patient was 42 years old and had multiple metabolic disturbances. Clinicians should maintain a high index of suspicion for FMD in men presenting with cryptogenic stroke, particularly when atherosclerotic burden is low or absent.

### 3.3 The diagnostic challenge: FMD coexisting with multiple metabolic disturbances – distinguishing causation from coincidence

A unique aspect of this case is the coexistence of FMD with multiple traditional vascular risk factors: hypertension, newly diagnosed diabetes, severe hypertriglyceridemia (17.54 mmol/L), hyperuricemia, and suspected nonalcoholic fatty liver disease. In a typical clinical setting, a middle-aged man with these risk factors presenting with a lacunar-type infarct might be attributed solely to small vessel disease or large artery atherosclerosis. However, the absence of significant atherosclerotic plaque on ultrasound and CTA, coupled with the classic angiographic appearance of FMD, pointed to FMD as the primary stroke etiology.

In this patient, the basal ganglia infarct location initially suggested hypertensive small-vessel disease. However, the presence of a unilateral, long-segment dysplastic lesion in the ipsilateral carotid artery, without significant stenosis elsewhere, and the exclusion of other embolic sources (e.g., atrial fibrillation, aortic arch pathology, or intracranial atherosclerosis) made artery-to-artery embolism from the FMD segment the most plausible mechanism. Moreover, the normal CRP and ESR, together with negative autoimmune workup, effectively ruled out active vasculitis, further strengthening the diagnosis of FMD. Nonetheless, we acknowledge that a direct causal link between FMD and the infarct cannot be definitively proven in a single case, and a coexisting contribution from microvascular disease is possible. Therefore, we advocate a balanced interpretation: FMD is the most likely primary etiology, but the stroke mechanism is probably multifactorial.

We also emphasize that the coexistence of multiple metabolic abnormalities in this patient is most likely coincidental rather than causative. There is currently no robust evidence to support a direct pathophysiological link between hypertriglyceridemia, hyperuricemia, or diabetes and FMD. The primary educational message of this case is not to propose a novel mechanistic association, but to serve as a clinical alert: the presence of a “metabolic storm” should not lower the index of suspicion for a rare vascular anomaly when the clinical or radiological findings (such as unilateral carotid beading) are atypical for a pure small-vessel or atherosclerotic disease.

This case serves as an important reminder that rare causes of stroke should be considered even when common risk factors are present—the two are not mutually exclusive. In fact, hypertension, a common comorbidity in FMD (present in 30%–60% of patients), may exacerbate the vascular damage caused by FMD and increase the risk of stroke (1). Although a direct pathophysiological link between metabolic abnormalities and FMD has not been established, emerging evidence suggests that shared mechanisms such as endothelial dysfunction and oxidative stress might influence the progression of both conditions—but these remain speculative and require further investigation.

### 3.4 Systemic nature of FMD and the importance of comprehensive screening

FMD is a systemic disease. Studies have consistently shown that among patients with carotid FMD, 58%–80% have involvement of at least one other vascular bed, most commonly the renal arteries (5, 11). The US Registry for FMD reported that 66% of patients with cerebrovascular FMD had multivessel involvement (12). This has important clinical implications: renal artery FMD can cause refractory hypertension and, rarely, renal impairment.

In our patient, renal artery duplex ultrasound at the 3-month follow-up revealed no evidence of renal FMD, which is reassuring. However, this finding does not diminish the importance of routine screening in all patients with cervicocranial FMD, as recommended by current consensus guidelines (3, 13). Even in the absence of clinical signs, asymptomatic renal FMD can be present and may progress over time; therefore, baseline imaging provides a valuable reference for future comparisons. Consistent with international recommendations, we performed renal artery screening. Given that duplex ultrasound is a screening tool rather than a definitive diagnostic method, renal artery CTA or MRA has been scheduled for the 1-year follow-up to definitively exclude renal artery involvement. Screening for iliac, mesenteric, or intracranial aneurysms was deferred to a scheduled 1-year follow-up, as the patient had no clinical indications (e.g., headache, abdominal bruit, or family history of aneurysms) that would mandate immediate invasive investigation.

### 3.5 Limitations of this report

Several limitations of this case report warrant acknowledgment. First, brain MRI with diffusion-weighted imaging (DWI), the gold standard for confirming acute infarction, could not be performed due to the patient's severe claustrophobia. This represents a significant constraint, as DWI would have provided detailed information on infarct topography, lesion age, and potential embolic patterns (e.g., multiple lesions suggestive of embolism). Although this limitation was partially mitigated by serial non-contrast CT scans performed at admission and 48 h after symptom onset—which clearly delineated the location and evolution of the left basal ganglia infarct, consistent with the patient's neurological deficits—we acknowledge that serial CT remains a suboptimal substitute for MRI, and the absence of DWI restricts the rigor of mechanistic interpretation. Second, comprehensive systemic vascular screening was not completed during the acute hospitalization; while renal artery duplex ultrasound was performed and showed no evidence of FMD, screening for involvement of other vascular beds (iliac, mesenteric, and intracranial arteries) was deferred to the scheduled 1-year follow-up, as the patient had no clinical indicators (e.g., headache, abdominal bruit, or family history of aneurysms) that would mandate immediate invasive investigation. Third, as a single case report, the findings may not be generalizable to the broader population of patients with FMD. Finally, long-term follow-up beyond three months is not yet available; nevertheless, the patient has demonstrated excellent short-term outcomes with complete neurological recovery (mRS 0), no recurrent events, and optimal control of blood pressure and metabolic parameters at the 3-month assessment. Continued prospective surveillance is planned to evaluate long-term prognosis at 1-year and 5-year follow-up intervals.

### 3.6 Treatment considerations

There are no randomized controlled trials specifically guiding the management of FMD-related stroke. Current recommendations are based on expert consensus and extrapolation from atherosclerotic stroke trials (14). For patients with FMD and ischemic stroke or TIA, antiplatelet therapy is generally recommended to reduce the risk of thromboembolic events. Our patient was started on dual antiplatelet therapy, which was intended as a short-term bridge (21 days) according to acute stroke guidelines, followed by lifelong single antiplatelet therapy, which aligns with current practice for non-cardioembolic stroke.

Aggressive risk factor modification is also warranted given the high prevalence of comorbid hypertension and metabolic abnormalities in this population. Blood pressure control is particularly important, as hypertension is both a risk factor for stroke and a potential exacerbator of FMD-related vascular damage (15). Statin therapy, while not specifically studied in FMD, is reasonable given the high rate of coexistent atherosclerosis and was primarily initiated to treat the patient's documented severe dyslipidemia. For patients with FMD and severe stenosis, dissection, or recurrent symptoms despite medical therapy, revascularization procedures may be considered, though data on long-term outcomes are limited.

## 4 Conclusion

This case report describes a 42-year-old man with acute ischemic stroke attributed to fibromuscular dysplasia of the internal carotid artery, confirmed by the pathognomonic “string-of-beads” sign on digital subtraction angiography. Several key messages for clinical practice emerge from this case. First, FMD should remain an active consideration in the differential diagnosis of ischemic stroke in middle-aged patients—including men—even when conventional cardiovascular risk factors are present, as the two are not mutually exclusive. Second, the classic “string-of-beads” angiographic appearance is diagnostic of the medial fibroplasia subtype, the most common form of FMD, and should prompt definitive vascular imaging. Third, given that FMD is a systemic disease, all patients with cervicocranial FMD should undergo screening of the renal and iliac arteries regardless of blood pressure status, as multivessel involvement is frequently encountered; the negative renal artery screening in our patient illustrates that FMD may remain isolated to the cervicocranial circulation, yet routine screening remains essential to detect occult involvement when present. Fourth, early recognition of FMD is critical for guiding appropriate secondary prevention strategies, including antiplatelet therapy and rigorous risk factor modification, to reduce the likelihood of recurrent cerebrovascular events.

We further emphasize that the coexistence of multiple metabolic abnormalities in this case is most likely coincidental, and the core educational value of this report lies in alerting clinicians to consider uncommon vascular pathologies even in patients with a heavy burden of traditional risk factors. Moreover, the diagnosis of FMD necessitates both the exclusion of vasculitis—supported by normal inflammatory markers and negative autoimmune serologies—and definitive vascular imaging; duplex ultrasound alone is insufficient to exclude renal artery involvement, and cross-sectional modalities such as CTA or MRA should be employed for comprehensive screening. Increased clinical awareness of this rare but treatable condition will facilitate earlier diagnosis and improved long-term outcomes.

## Patient perspective

**“**When I first had the stroke, I was terrified. I'm only 42 and thought I was healthy. Finding out I had this rare condition alongside diabetes and high cholesterol was overwhelming. The doctors explained everything clearly, and I'm committed to taking my medications and coming for check-ups. I feel completely back to normal now and am very grateful to the medical team. Knowing that my kidney arteries are clear at the 3-month check-up was a huge relief.” (Paraphrased from an interview with the patient, with his consent).

## Data Availability

The datasets presented in this article are not readily available because the dataset generated for this case report is derived from routine clinical care and contains potentially identifiable patient information. Due to patient privacy protection regulations and the limitations of the informed consent obtained, the raw data (including full medical records and original imaging files) cannot be made publicly available or shared. All anonymized clinical data relevant to the case are presented within the manuscript. Requests to access the datasets should be directed to Yinbao Hu, yinbaohu@hotmail.com.
